# Activating Relatives to Get Involved in Care After Surgery: Protocol for a Prospective Cohort Study

**DOI:** 10.2196/38028

**Published:** 2023-01-06

**Authors:** Selma C W Musters, Sani Kreca, Susan van Dieren, Hanneke van der Wal-Huisman, Johannes A Romijn, Wendy Chaboyer, Els J M Nieveen van Dijkum, Anne M Eskes, ARTIS Consortium

**Affiliations:** 1 Department of Surgery Amsterdam UMC, Cancer Center Amsterdam University of Amsterdam Amsterdam Netherlands; 2 Department of Surgery University of Groningen University Medical Center Groningen Groningen Netherlands; 3 Amsterdam UMC University of Amsterdam Amsterdam Netherlands; 4 Menzies Health Institute Queensland and School of Nursing and Midwifery Griffith University Gold Coast Australia; 5 Department of Surgery Amsterdam UMC, Amsterdam Gastroenterology Endocrinology Metabolism and Cancer Center Amsterdam University of Amsterdam, Amsterdam Netherlands; 6 See Acknowledgements

**Keywords:** family-centered care, postoperative, surgical oncology, cohort, prospective study

## Abstract

**Background:**

Postoperative complications and readmissions to hospital are factors known to negatively influence the short- and long-term quality of life of patients with gastrointestinal cancer. Active family involvement in activities, such as fundamental care activities, has the potential to improve the quality of health care. However, there is a lack of evidence regarding the relationship between active family involvement and outcomes in patients with gastrointestinal cancer after surgery.

**Objective:**

This protocol aims to evaluate the effect of a family involvement program (FIP) on unplanned readmissions of adult patients undergoing surgery for malignant gastrointestinal tumors. Furthermore, the study aims to evaluate the effect of the FIP on family caregiver (FC) burden and their well-being and the fidelity of the FIP.

**Methods:**

This cohort study will be conducted in 2 academic hospitals in the Netherlands. The FIP will be offered to adult patients and their FCs. Patients are scheduled for oncological gastrointestinal surgery and have an expected hospital stay of at least 5 days after surgery. FCs must be willing to participate in fundamental care activities during hospitalization and after discharge. Consenting patients and their families will choose to either participate in the FIP or be included in the usual care group. According to the power calculation, we will recruit 150 patients and families in the FIP group and 150 in the usual care group. The intervention group will receive the FIP that consists of information, shared goal setting, task-oriented training, participation in fundamental care, presence of FCs during ward rounds, and rooming-in for at least 8 hours a day. Patients in the comparison group will receive usual postoperative care. The primary outcome measure is the number of unplanned readmissions up to 30 days after surgery. Several secondary outcomes will be collected, that is, total number of complications (sensitive to fundamental care activities) at 30 and 90 days after surgery, emergency department visits, intensive care unit admissions up to 30 and 90 days after surgery, hospital length of stay, patients’ quality of life, and the amount of home care needed after discharge. FC outcomes are caregiver burden and well-being up to 90 days after participating in the FIP. To evaluate fidelity, we will check whether the FIP is executed as intended. Univariable regression and multivariable regression analyses will be conducted.

**Results:**

The first participant was enrolled in April 2019. The follow-up period of the last participant ended in May 2022. The study was funded by an unrestricted grant of the University hospital in 2018. We aim to publish the results in 2023.

**Conclusions:**

This study will provide evidence on outcomes from a FIP and will provide health care professionals practical tools for family involvement in the oncological surgical care setting.

**International Registered Report Identifier (IRRID):**

DERR1-10.2196/38028

## Introduction

### Background

Postoperative complications and hospital readmissions are known to negatively influence the short- and long-term quality of life (QoL) of patients [1]. In addition, complications and readmissions also increase health care demands and costs [2,3]. Readmission rates after gastrointestinal surgery increase up to 30% [4] and are higher compared with general surgery in that these rates range from approximately 4% to 11% [5,6]. In addition, complication rates after gastrointestinal surgery are 34% [7], which is approximately 3 to 8 times higher compared with general surgery [8,9]. Frequent complications in this patient population include delirium, pneumonia, and wound infections [7]. Reducing these readmissions and postoperative complications in patients undergoing oncological gastrointestinal surgery is a priority.

Some postoperative complications are potentially preventable [10]. Several studies have shown that optimizing basic care by executing fundamental care activities prevents postoperative complications, such as pneumonia, urinary tract infections, and delirium [10-12]. Fundamental care activities include early mobilization, breathing exercises, dental hygiene, orientation in time and place, and eating together [13]. Although fundamental care seems simple, it is prone to be missed care [13,14].

The authors of a systematic review of 18 studies concluded that up to 75% of nurses omitted some kind of fundamental care tasks [15]. In a recently published meta-review of 7 systematic reviews, the following categories of missed nursing care were identified: (1) fundamental physical care; (2) communication and information sharing with patients and families; (3) self-management, autonomy, and education of patients and families, including care planning, discharge planning, and decisions; and (4) emotional and psychological care, including spiritual support [14]. Poorly executed or missing aspects of this care threaten patients’ QoL and satisfaction, resulting in higher numbers of adverse outcomes and poor care experiences [13,14].

After hospital discharge, family caregivers often play an important role in the delivery of fundamental care activities to patients with cancer [16]. However, family caregivers often feel unprepared to deliver proper care of their loved ones after discharge, and they also feel that they lack the necessary skills and knowledge [17]. This might be because family caregivers are often considered visitors during patients’ hospital stay and are sidetracked [18]. Thus, family caregivers are not normally actively involved in postoperative care during hospitalization.

Despite the uncertainty that family caregivers experience, in the past few decades, family-centered care (FCC) has been shown to potentially improve the quality of health care [13,19-21]. Family caregivers have become important care partners who are able to participate in all aspects of care delivery [22,23]. For instance, studies in which family caregivers were actively involved in performing fundamental care activities during hospital admission have shown a reduction in postoperative complications such as delirium and pneumonia [12,24]. The authors of a review of systematic reviews concluded that FCC could be an effective approach for improving the quality of health care, but there was a lack of high-quality studies investigating the effectiveness of FCC [25].

Family caregivers might be better prepared to deliver adequate care to their loved ones after discharge if they are involved in fundamental care during hospitalization, which could also lead to improved adherence to these care activities. Therefore, the incidence of complications and related readmissions might decrease. To promote active family involvement, we developed a multicomponent family involvement program (FIP) in which family caregivers could be actively involved in caring for their loved ones during hospitalization after oncological gastrointestinal surgery [26]. In a pilot study, our FIP was shown to be feasible for patients, family caregivers, and health care professionals [16,27]. In addition, family caregivers had a better understanding and knowledge of their loved one’s recovery process [27].

### Objectives

Although the results of our pilot study on the FIP were promising, the lack of evidence on the relationship between family involvement and outcomes in patients with gastrointestinal cancer after surgery points to the need for a prospective cohort study. Therefore, the main objective of this study is to evaluate whether FIP for adult patients undergoing oncological gastrointestinal surgery reduces the number of unplanned readmissions compared with usual care. The secondary aims are to evaluate the effect of the FIP on family caregivers by assessing caregiver burden and family caregivers’ well-being and to assess the fidelity of the FIP. Here, we describe the study design and summarize the study protocol, which may also provide practical tools for health care professionals to adopt a more family-centered approach.

## Methods

### Study Design

This study is known by the acronym “ARTIS,” which stands for Activating Relatives to Get Involved in Care After Surgery. The ARTIS study is a patient preference multicenter prospective cohort study. The study protocol described here is reported according to the applicable criteria of the Standard Protocol Items: Recommendations for Interventional Trials (SPIRIT) [28] and the Template for Intervention Description and Replication (TIDieR) [29]. The results of this study will be reported according to the Strengthening the Reporting of Observational Studies (STROBE) [30].

### Study Setting

The study will be conducted at 5 surgical wards that provide care to patients after oncological gastrointestinal surgery in two Dutch tertiary university hospitals in the Netherlands: (1) Amsterdam University Medical Center (UMC), location Academic Medical Center (AMC) and Vrije Universiteit Medical Center, Amsterdam, and (2) University Medical Center Groningen (UMCG), Groningen. The average number of patients undergoing oncological gastrointestinal surgery as treatment for malignant pancreatic, gastric, esophageal, liver, colorectal, or peritoneal tumors in Amsterdam UMC is 415 per year and in UMCG 330 per year. Each participating surgical ward has 25 to 30 beds with a nursing:patient ratio of either 1:4 or 1:5. The participating wards were chosen because health care professionals expressed a willingness to adopt a more family-centered approach to their care.

### Eligibility Criteria

#### Patients

In this study, we will include adult patients (≥18 years) scheduled for gastrointestinal surgery as treatment for malignant pancreatic, stomach, esophageal, liver, colorectal, or peritoneal tumors. The expected hospital stay must be at least 5 days after the operation, which makes adequate training and coaching of family caregivers under the supervision of nurses possible. Furthermore, patients will have to be able to read and write the Dutch language and will be included after providing written consent. Patients will be excluded if they remain in the intensive care unit (ICU) for >72 hours directly after surgery, as family caregivers do not have the opportunity to be involved in care in the first 5 days after the operation. In addition, patients will be excluded if the tumor is considered unresectable during surgery, as these patients do not receive the intended surgery and are discharged after one day.

#### Family Caregivers

The eligibility criteria for adult family caregivers are that they are willing and able to deliver fundamental care during hospitalization (for a minimum of 8 hours per day) and after discharge (if needed). Furthermore, they should be nominated as the appointed family caregiver by the patient and should not require support from health care professionals to carry out their own self-care activities. Furthermore, family caregivers will have to be able to read and write the Dutch language and will be included after providing written consent. In this study, family caregivers could be relatives or close friends who know the patient and either live with or are involved in the care of the patient. They are individuals who provide support and with whom the patients have a significant relationship.

### Intervention

#### Overview

The FIP is a multicomponent intervention developed using a multiphase quality improvement design [26]. The main components of the program are as follows: (1) active involvement of family caregivers and (2) training and coaching of health care professionals in the FCC. The development process and rationale of this program have been described in further detail by Eskes et al [26]. The FIP is performed in addition to the usual postoperative care.

#### Component 1: Active Involvement of Family Caregivers

For the first component, multiple tasks involving families in fundamental care activities are described.

##### Invite Family Caregivers to Participate in Fundamental Care Activities

To standardize FIP delivery, family caregivers will be asked to participate in evidence-based fundamental care activities (Textbox 1). In addition, family caregivers can participate in optional care activities (Textbox 2).

**Fundamental care activities executed by family caregivers.**

- Three times a day
  - Mobilization defined as getting a patient out of bed (ie, sitting out of bed and ambulation) [12,31-33]
  - Encouraging oral intake and companionship during meals (ie, breakfast, lunch, and dinner) and ensuring that patients are seated at the table when meals are served [11,32]
  - Supporting patients in doing breathing exercises (ie, coughing and deep breathing) [12,34,35]
  - Supporting active orientation; specific time-, place-, and person-related information in the context of the present day; and daily discussions on actual items (eg, news) [31,32]
- Two times a day
  - Supporting in carrying out oral care (brushing teeth and using mouthwash) [12,34,35]

**Optional care activities executed by family caregivers.**

- General care
  Washing and dressing of the patient
  Assistance with the use of the toilet
- Wound-related care
  Wound dressing
- Administering medication
  Injecting anticoagulation
- Drain-related care
  Taking care of abdominal drains
  Taking care of nasogastric tubes

##### Information About Fundamental Care Activities

Family caregivers who participate in the FIP will gain access to a mobile app (so-called “Mantelzorgapp”) before patients’ hospital admission. The app has detailed descriptions of the following: (1) the rationale for performing the fundamental care activity, (2) preparation for the activity, (3) the needed material for the activity, (4) a step-by-step description on how to perform the activity, and (5) time and frequency of performing the activity. The app also includes a daily diary in which the family caregivers tick boxes if they have performed a fundamental care activity (yes or no) with writing space for comments. Furthermore, the app includes discharge information, such as whom to contact if they have concerns about patients’ health condition. The app can be downloaded without any cost from the Apple Store (Apple Inc) and Google Play Store (Google LLC) and is suitable for several mobile devices (ie, phones and tablets). If family caregivers are unable to use the app, they will receive paper-based information.

##### Shared Goal Setting With the Patient, Family Caregiver, and Nurse

On the basis of mutual agreement among the patient, the family caregiver, and the nurse, a personalized plan for the patient and family caregiver will be made within 24 hours after the surgery. This plan includes the patient’s care needs and personal goals of the family caregiver. This plan will be documented in the electronic patient record. Active participation will start on the first day after surgery in the ward (or within 72 hours in case of ICU admission) and will be continued for at least 5 days after surgery. After this period, the family caregivers choose whether or not to continue the program. We plan to work with the same family caregiver throughout the study period. However, if there are multiple family caregivers, we will recruit a maximum of 3 family caregivers who are available and interested. These family caregivers need to alternate during admission, and each family caregiver needs to be involved in the FIP for a minimum of one day.

##### Task-Oriented Training of Family Caregivers to Deliver Fundamental Care Activities

During the FIP, the family caregiver will receive training from a registered nurse. All the activities will be supervised by the nurse until the family caregiver is competent, as determined by the nurse, to carry out the activities on their own. To facilitate adequate care delivery, the objective of each activity, including instructions on how to deliver the activity, is described in full detail and accessible in the app. If the activity is carried out, the family caregiver registers it on the registration form in the app. Additional activities will also be recorded in the app.

##### Physical Proximity (Rooming-in)

Family caregivers are asked to stay with the patient for at least 8 hours per day during the first 5 postoperative days, which offers the opportunity to learn and become competent in new fundamental care activities under supervision. As most of these activities should be undertaken 3 times a day, presence of at least 8 hours provides them with the opportunity to complete these activities.

##### Presence of Family Caregivers During Medical Ward Rounds

Family caregivers are encouraged to be present and to be actively involved during medical rounds. Health care professionals (eg, doctors and nurses) will support the family caregivers throughout the hospital stay, answer questions related to the FIP, and identify unmet needs.

#### Component 2: Training and Coaching of Health Care Professionals

To support the delivery of the FIP, structured training sessions will be given to doctors and nurses. The training will mainly focus on the four core concepts of FCC: (1) dignity and respect, (2) information sharing, (3) participation, and (4) collaboration [26]. In the session, the rationale, goals, and benefits of the FIP and the concept of passive and active family involvement will also be explained. In addition, quality- and safety-related topics will be discussed. The training session will be delivered by one of the members of the research team.

### Usual Postoperative Care

Patients in the usual care group will receive care based on the Enhanced Recovery After Surgery principles, which include early mobilization, early postoperative feeding, goal-directed fluid therapy, and nonnarcotic analgesia [36]. In addition, patient engagement and a multidisciplinary team approach to the usual postoperative care will be used [36]. Patients will receive care pathways for their specific type of surgery in which day-to-day targets are provided, which promotes autonomy and cooperation from the patients. Patients are encouraged to actively mobilize from the day of surgery until hospital discharge (ie, stay out of bed for 2 hours on the day of surgery and at least 6 hours per day from the first postoperative day until hospital discharge). The patients will not receive detailed information about the content of the FIP. No rooming-in opportunities will be offered to the usual care group, except for family visits (ie, a maximum of 2 visitors at the patient’s bedside between 11 AM and 8 PM will be permitted).

### Outcomes

The primary patient outcome is the number of unplanned readmissions up to 30 days after surgery (Table 1). Secondary outcomes include the total number of complications at 30 and 90 days after surgery and the number of complications sensitive to fundamental care activities (eg, pneumonia, urinary tract infection, wound infection, deep vein thrombosis, malnutrition, and delirium) at 30 and 90 days after surgery. These data will be collected from patient records, and complications will be classified according to the revised version of the Clavien-Dindo classification system [37]. Secondary outcomes also include emergency department visits, ICU admissions up to 30 and 90 days after surgery, and hospital length of stay. Furthermore, patients’ QoL will be measured using the EQ-5D-5L [38], and the level of anxiety and depression in patients will be measured using the Hospital Anxiety and Depression Scale (HADS; Table 1) [39,40]. Patient satisfaction with care and sleep quality will be measured using the numeric rating scale (NRS) ranging from 0 to 10, with higher scores indicating higher satisfaction or sleep quality (Table 1). Sleep quality will be assessed once a day, from the first day after surgery until 5 days after the operation. In addition, the amount of home care (in hours per week) after discharge will be measured (Table 1). The risk screening scores routinely measured during hospital admission will be collected, ie, the Short Nutritional Assessment Questionnaire score (SNAQ) [41], the Delirium Observation Scale score (DOS) [42], the Braden scale [43], the Amsterdam UMC Extension of the John Hopkins Highest Level of Mobility scale score (AMEXO) [44] and the John Hopkins Fall Risk Assessment (JHFRAT; Table 1) [45].

In addition to the effect of the FIP on patient outcomes, we measured the effect of the FIP on family caregiver outcomes, including caregiver burden and well-being up to 90 days after participating in the FIP (Table 1). The Valuation of Informal Care Questionnaire (iVICQ) measures objective burden using the following items: duration and intensity of informal care, patient health, need for permanent surveillance, patients’ living situations, and use of professional care [46]. Subjective burden will be measured using the Caregiver Strain Index plus (CSI+) [47], self-rated burden scale [48], and perseverance time [49]. The well-being of family caregivers will be measured using the Care-related Quality of Life instrument (CarerQoL-7D) and Care-related Quality of Life–Visual Analog Scale (CarerQoL-VAS; Table 1) [50]. Finally, the costs of the FIP will also be measured using the iVICQ [46].

To measure adherence, which is considered a bottom-line measurement of intervention fidelity [51], we will record how many patients are offered the FIP and how many patients decline, including the reasons if they want to disclose. We will use the data recorded by family caregivers to assess the fidelity of the intervention. Up to 5 days after surgery, family caregivers will be asked to record the fundamental and optional care activities they have carried out in the app, and if not completed, to add a reason. The family caregiver registers the activity on the registration form of the app. If multiple family caregivers participate in the FIP, they will all be asked to record the executed activities. Rooming-in of the family caregivers during admission will be checked by ward nurses and researchers (SM, SK, and HWH).

**Table 1:** Outcome variables for patients and their family caregivers.

					T0, baseline		T1, during 5 days after the operation			T2, at discharge		T3, discharge+30 days		T4, discharge+90 days
**Patient**														
		Unplanned readmissions^a^										✓		✓
		Complications (sensitive to fundamental care activities)^a,b,c^										✓		✓
		First aid visit^a^										✓		✓
		ICU^d^ admission^a^										✓		✓
		EQ-5D-5L			✓				✓			✓		✓
		HADS^e^			✓				✓			✓		✓
		NRS^f^ patient satisfaction							✓					
		NRS sleep quality					✓							
		LOS^g^							✓					
		Bodyweight (in kilogram)			✓				✓			✓		✓
		Professional home care (indications)							✓			✓		✓
		SNAQ^h^			✓							✓		✓
		Braden scale			✓							✓		✓
		DOS^i^			✓		✓					✓		✓
		AMEXO^j^					✓					✓		✓
		JHFRAT^k^			✓							✓		✓
**Family caregiver**														
	**Objective burden**													
			Duration of informal care	✓				✓			✓		✓	
			Intensity of informal care	✓				✓			✓		✓	
			Health of patient	✓				✓			✓		✓	
			Need for permanent surveillance	✓				✓			✓		✓	
			Patients’ living situation	✓				✓			✓		✓	
			Use of professional care	✓				✓			✓		✓	
	**Subjective burden**													
			CSI+^l^	✓				✓			✓		✓	
			Self-rated burden scale	✓				✓			✓		✓	
			Perseverance time					✓			✓		✓	
	**Well-being**													
			CarerQoL-7D^m,n^	✓				✓			✓		✓	

^a^Unplanned readmissions, complications, first aid visits, and ICU admissions will be measured 30 and 90 days after surgery.

^b^Complications will be classified according to the revised version of the Clavien-Dino classification system.

^c^Complications sensitive to fundamental care activities: pneumonia, urinary tract infection, wound infection, deep vein thrombosis, malnutrition, and delirium.

^d^ICU: intensive care unit.

^e^HADS: Hospital Anxiety and Depression Scale.

^f^NRS: numeric rating scale.

^g^LOS: length of stay.

^h^SNAQ: Short Nutritional Assessment Questionnaire score.

^i^DOS: delirium observation scale.

^j^AMEXO: Amsterdam UMC Extension of the John Hopkins Highest Level of Mobility scale score.

^k^JHFRAT: John Hopkins Fall Risk Assessment.

^l^CSI+: Caregiver Strain Index plus.

^m^CarerQoL-7D: Care-related Quality of Life.

^n^CarerQoL-VAS: Care-related Quality of Life–Visual Analog Scale.

### Study Procedure

During the preoperative outpatient clinic visit, the patients and their family caregivers will be screened for eligibility and informed about the study (Table 2). If they are interested, a researcher (SM, SK, or HWH) will contact the patients after the visit to determine their preference for the study arm. If patients prefer the intervention arm, at least 2 days before hospital admission, the researcher will confirm the patients’ participation depending on the ward capacity. The FIP starts <72 hours after surgery when patients are transferred from the postanesthesia care unit to the participating surgical wards. Depending on the ward capacity and patient preferences, the program can be continued until discharge. During the follow-up period (ie, 30 and 90 days after surgery), the patient and family caregiver will be contacted by telephone by SM, SK, or HWH and asked to complete the questionnaires.

**Table 2:** Study procedure.

Timing			Activity		
**Prehospital admission^a^**					
	Outpatient clinic visit	Recruitment of patients and family caregivers, screening for eligibility, and informed consent procedure Group allocation based on patient and family preferences			
	1 week before hospital admission	Patients and family caregivers from the intervention group will get access to the mobile app (so-called Mantelzorgapp)			
	2 days before admission	The researcher will confirm the patients’ participation in the intervention group depending on the ward capacity			
**Clinical phase^b^**					
	Within 24 hours after surgery	Family caregivers will receive a personalized plan based on the identified patients’ care needs, and the plan includes the personal goals of the family caregiver Goals will be documented in the electronic patient record Patients and family caregivers will complete questionnaires about their baseline characteristics			
	Day of surgery until 5 days after operation	Family caregivers start by participating in the fundamental care activities (and optional care activities) Family caregivers will register all activities in the app. Depending on the ward’s capacity and patients’ preferences, the program could be continued until discharge			
	At discharge	Patients and family caregivers will complete discharge questionnaires			
**After discharge**					
	Follow-up 30 days and 90 days after discharge	The researcher will send questionnaires to patients and family caregivers for measuring outcomes The researcher will contact the patient and family caregiver by telephone for outcome measures not mentioned in the questionnaires The researcher will collect data on the number of readmissions and complications as registered in the electronic patient record			

^a^Information about the FIP.

^b^The required clinical data regarding baseline characteristics and outcomes will be collected during hospitalization using standardized case report forms.

### Allocation

Participation in the FIP is based on the preferences of patients and, if available, their family caregivers. This means that when the patients and their relatives want to participate in the program, they will be allocated to the intervention group and when the patients or their relatives do not want to participate in the program (but give consent for observation in the usual care group), they will be allocated to the usual care group. By using this patient preference study design, we might introduce contamination between the intervention and comparison groups, as patients in the comparison group might learn about fundamental care activities and adopt them themselves or receive informal care without the program. Nevertheless, this study design was selected deliberately because no clear signs of contamination were observed between the study groups in the pilot study [16]. To minimize the risk of between-group contamination, the information for patients and their families before inclusion will not include detailed information about the fundamental care activities that comprise the program.

### Sample Size

The sample size is based on the primary objective of evaluating the effects of the FIP on unplanned readmissions. In our pilot study, we found an absolute reduction of unplanned readmission rates of 15% [16] and considered this 15% reduction of unplanned readmissions as clinically relevant, representing a readmission rate of 20% in the intervention group and 35% in the usual care group. To detect a 15% reduction in readmission rates at a 5% significance level (α) and a power of 80% (1 – β), a sample size of 136 patients per group is necessary, using a continuity-corrected chi-square test. We assume a 10% to 20% loss to follow-up (ie, not completing the questionnaires), and to compensate for this, the total sample size per group will be at least 150. Loss to follow-up is expected owing to the use of repeated questionnaires, which may burden patients. Recruitment ends as soon as both the groups have included 150 patients. To minimize attrition, we will collect detailed contact information and offer patients alternative data collection methods such as paper-based questionnaires if patients do not have an email address [52].

### Data Collection Methods

#### Patients

At baseline, the following patient characteristics will be collected from the patient records: sex [53], age [53], length in cm, weight at admission in kg [54], BMI in kg/m^2^ [54], WHO performance status [55], American Society of Anesthesiologists Physical Status Classification (ASA PS Classification) [55], use of ≥5 medicaments [56], preoperative diagnosis, tumor type, type of surgery (ie, open, minimally invasive, or robot-assisted), and type of resection. In addition, the risk screening scores (ie, SNAQ, DOS scale, Braden scale, AMEXO, and JHFRAT) will be collected on the day of hospital admission. The number of days spent in the postanesthesia care unit will be collected from the patient records.

Furthermore, socioeconomic data will be collected via a patient questionnaire on the day of admission, including the highest level of formal education, marital status, number of children (≤18 years), and number of live-in children, hours per week of unpaid work and paid work [53]. The degree of social isolation before admission could be a potential confounder, as it is known that socially isolated patients have worse outcomes [57] and will therefore be measured using the Friendship scale [58].

#### Family Caregivers

On the day of admission, baseline characteristics of the family caregivers will be collected using the iVICQ [46]. Background characteristics include sex, age, educational level, relationship between family caregiver and patient, household composition, unpaid and paid work, monthly net household income, and health status.

### Data Management

Patient data will be entered into a web-based data management system (Castor Electronic Data Capture, version 2022.4.1.3), which complies with all applicable laws and regulations. Family caregivers’ data will be entered into an online protected database connected to the app. If family caregivers are unable to enter their data into the app, we will provide written case report forms. Participants are assigned a study number but can be identified in the database by their ID number. The participation identification log will be kept separate from the patient data and will be safeguarded by the principal investigator. For the participating centers, a separate site-specific subject identification log will be kept at each study site.

The integrity of both patients’ and family caregivers’ data will be protected through a variety of mechanisms. Valid rules, range checks, and consistency checks against stored data will be supported. Access to the study data will be restricted, and a password will be used to control access. For the online database, 2-faced authentication is needed for access. Access to the app for family caregivers is password protected, which prevents the user data from being revealed if the device is stolen. Castor data will be stored on certified servers (ISO 27001, ISO 9001, and NEN 7510) as well as app data (ISO 27001 and NEN 7510). Data will also be stored locally in the department’s computer system drive. All hard-copy original study forms will be stored in a secure and accessible place. Files will be stored for 15 years after completion of the study. This study will not be monitored because the potential risk to patients participating in the study is negligible.

### Statistical Methods

#### Overview

Statistical analyses will be performed using the R statistical software package (version 3.6.2; R Foundation for Statistical Computing). Descriptive statistics will be used to summarize the baseline characteristics and process outcomes of the patients and family caregivers. Continuous variables will be presented as means and SDs or medians and IQRs according to the distribution of the variables. Categorical variables will be presented as counts and percentages (%), and numbers and frequencies will be presented for dichotomous data. The distribution of variables will be determined by inspecting histograms and box plots.

The patients’ baseline characteristics of the study arms will be tested for statistically significant differences using the chi-square statistics, Mann-Whitney *U* test, independent samples 2-tailed *t* test, or Fisher exact test. If there are differences between the FIP and treatment groups, we will adjust for confounding variables using multivariate regression analyses.

#### Outcomes

Univariable regression and multivariable regression analyses will be conducted using stepwise backward selection. For the primary outcome (ie, unplanned readmissions up to 30 days after surgery), we will conduct logistic regression analyses. For continuous secondary outcomes, we will use linear regression analyses; for dichotomous outcomes, we will use logistic regression analysis. We will use a general linear mixed model to analyze differences in sleep quality, DOS scores, and AMEXO scores over 5 days after the operation (Table 1). If a difference is observed, a post hoc power analysis will be used to examine which time point differs significantly. A 2-sided *P* value ≤.05 will be considered statistically significant. A 95% CI for the beta coefficient (β) will be calculated.

#### Additional Analyses

In this study, some additional analyses will be performed. We will analyze whether performing one or more optional care activities (Textbox 2) and the involvement of multiple family caregivers per patient can modify patient outcomes. Furthermore, in a sensitivity analysis, we will compare outcomes from patients in the FIP study arm to those from the comparison group whose family caregivers decline to participate in the FIP. The analysis will be based on intention-to-treat principles, meaning that every patient and family caregiver will be analyzed in the group that they chose before surgery.

#### Handling of Missing Data

If >40% of the missing data in the data set occur, multiple imputation by the chained equations method in R will be used [59,60]. Overall, 5 independent copies of the data will be analyzed. The estimates of the variables will be pooled according to Rubin rules. The pooled analysis will be presented. A complete case analysis will be performed as sensitivity analyses [60]. If >40% of the data are missing, we will use the observed data and report the extent of the missing data [59].

### Ethics Approval

The Medical Ethics Review Committee (METC) of the Amsterdam UMC (location AMC), Amsterdam, the Netherlands, approved the study. The METC reviewed the study protocol and concluded that the Medical Research Involving Human Subject Act did not apply to this study (reference number METC: W19_497#20.015). Written informed consent will be obtained from all the participants.

## Results

The first participant in this study was enrolled in April 2019 at the Amsterdam UMC, AMC. The follow-up period of the last participant ended in May 2022. The study was funded by an unrestricted grant of the University hospital in 2018. We aim to publish the results at the end of 2023.

## Discussion

### Principal Findings

Currently, FCC is becoming more widespread in hospital environments [25]. However, there is still a lack of rigorous evidence regarding the effects of FCC interventions and programs [19]. This multicenter cohort study allows us to evaluate the effect and process of an evidence-based multicomponent FIP in comparison with the usual postoperative care in patients undergoing oncological gastrointestinal surgery. We hypothesize that the number of unplanned readmissions and complications could be reduced by the FIP without increasing caregiver burden.

Before we planned this substantial evaluation of the FIP, we followed a stepwise approach guided by the Medical Research Council framework, as the FIP can be considered a complex intervention [61]. The development phase of the FIP began in 2015. First, we developed an evidence-based and theoretically grounded program [26]. This phase was followed by testing program feasibility in a pilot study, which gave us input about the required sample size [16]. In addition, we evaluated the experiences of family caregivers who participated in the FIP in a qualitative study [27]. The results of the second phase were used to develop the intervention study in which we aimed to assess the effectiveness of the FIP. We initiated a randomized controlled trial (RCT; Dutch Trial Register: NL66712.018.18). However, after consenting, many patients and families mentioned a strong preference and withdrew from both groups directly after randomization, stating in their reasoning that they either be involved as participating family caregiver or not. Continuing the trial would have probably led to high dropout rates and, therefore, decreased the generalizability of results to clinical practice [62].

To deal with the strong preferences of patients and their families, we considered changing the design to a patient preference trial. These trials have been introduced to give patients the opportunity to participate in the study arm they prefer [63,64] and have the main advantage of a lower risk of bias, especially in subjective outcome measures, and a decrease in dropout rates [64]. A consequence of choosing this study design is that it requires a very large sample size when a considerable number of patients have a preference for one particular study arm [64] and that it still needs a group of patients (and families) without preferences who want to be randomized, which appeared not to be the case in our study. Therefore, the best available option was to change the design to a patient preference observational cohort study, which is considered a valuable alternative when an RCT does not meet patients’ preferences [65].

Nevertheless, we realize that this patient preference study design still influences the external validity. Eligible patients without an available family caregiver cannot participate in the FIP, and most probably, a substantial number of patients need to decline this opportunity. On the basis of our previous work, we expect that this will be the case in approximately 20% of the patients and another 15% of the family caregivers are expected to decline participation [16]. Therefore, it is essential to compare as many prognostic variables as possible between the 2 groups to assess baseline comparability, including patients’ perceived social isolation and living status [66].

### Implementation of FIP

By conducting this study, we will gain a better understanding of the behaviors of patients, families, and health care professionals that need to be changed and which implementation strategies are valuable to achieve this change. This input is needed to implement the results after finishing our cohort study and to guide other organizations also in implementing the FIP. In the pilot study, we developed an implementation plan using Grol and Wensing model [67] that facilitates changes in health care using a stepwise approach. On the basis of the implementation recommendations derived from our pilot study, we implemented our FIP in this cohort study. A summary of our implementation recommendations and lessons learned were published in the article by Schreuder et al [16]. An understanding of how the FIP was implemented by assessing the adherence to the FIP, the bottom-line measurement of intervention fidelity [51], will provide valuable insights when interpreting the effects of the FIP [51].

A potential challenge for implementing the FIP is that it requires health care professionals to accept an FCC approach [21]. This is considered a challenge because some health care professionals may not yet regularly engage patients’ family caregivers in their daily practice [21]. Therefore, training and coaching health care professionals in the FCC is an essential component of FIP. Based on our pilot study, acceptable levels of time investment by doctors and nurses were found, and for most nurses, the workload was reduced to some extent [16]. Furthermore, most doctors value the presence of family caregivers during medical ward rounds [16]. Because of the positive experiences of health care professionals during the pilot phase of the study, we did not investigate their experiences again in this study. Nevertheless, one should be aware that work role conflicts because of having family caregivers involved in care are associated with nurses’ distress and impaired quality of care [68]. In addition, one should be aware that some patients and health care professionals might view the FIP as an economical solution, replacing nurses with family caregivers rather than trying to improve health care [69,70].

### Conclusions

In conclusion, this study will provide health care professionals important information about the relationship between a multicomponent FIP and outcomes in a broad group of patients with cancer undergoing surgery and their family caregivers. The FIP, which consists of evidence-based fundamental care activities for family caregivers, will provide practical tools for health care professionals to adopt a more family-centered approach. Future research should focus on the implementation strategies of FCC, choosing study designs that deal with patient preferences in complex interventions and involving patients when designing studies.

## Abbreviations

AMC: Academic Medical Center
AMEXO: Amsterdam UMC Extension of the John Hopkins Highest Level of Mobility Scale
ARTIS: Activating Relatives to Get Involved in Care After Surgery
ASA PS Classification: American Society of Anesthesiologists Physical Status Classification
CarerQoL-7D: Care-related Quality of Life instrument
CarerQoL-VAS: Care-related Quality of Life–Visual Analog Scale
CSI+: Caregiver Strain Index plus
DOS: Delirium Observation Scale
FC: family caregiver
FCC: family-centered care
FIP: family involvement program
HADS: Hospital Anxiety and Depression Scale
ICU: intensive care unit
iVICQ: Valuation of Informal Care Questionnaire
JHFRAT: John Hopkins Fall Risk Assessment
NRS: numeric rating scale
QoL: quality of life
SNAQ: Short Nutritional Assessment Questionnaire score
SPIRIT: Standard Protocol Items: Recommendations for Interventional Trials
STROBE: Strengthening the Reporting of Observational Studies
TIDieR: Template for Intervention Description and Replication
UMC: University Medical Center
UMCG: University Medical Center Groningen

## Data Availability

The data sets generated and analyzed during this study are not publicly available because they involve sensitive human research participant data but are available from the corresponding author upon reasonable request.
